# A Randomized, Controlled, Trial of Short Cycle Intermittent Compared to Continuous Antiretroviral Therapy for the Treatment of HIV Infection in Uganda

**DOI:** 10.1371/journal.pone.0010307

**Published:** 2010-04-22

**Authors:** Steven J. Reynolds, Cissy Kityo, Claire W. Hallahan, Geoffrey Kabuye, Diana Atwiine, Frank Mbamanya, Francis Ssali, Robin Dewar, Marybeth Daucher, Richard T. Davey, Peter Mugyenyi, Anthony S. Fauci, Thomas C. Quinn, Mark R. Dybul

**Affiliations:** 1 National Institute of Allergy and Infectious Diseases, National Institutes of Health, Bethesda, Maryland, United States of America; 2 Johns Hopkins University School of Medicine, Baltimore, Maryland, United States of America; 3 Joint Clinical Research Center, Kampala, Uganda; 4 SAIC-Frederick, Inc., National Cancer Institute, National Institutes of Health, Bethesda, Maryland, United States of America; 5 O'Neill Institute for National and Global Health Law, Georgetown University Law Center, Washington, D. C., United States of America; 6 George W. Bush Institute, Dallas, Texas, United States of America; University of New South Wales, Australia

## Abstract

**Background:**

Short cycle treatment interruption could reduce toxicity and drug costs and contribute to further expansion of antiretroviral therapy (ART) programs.

**Methods:**

A 72 week, non-inferiority trial enrolled one hundred forty six HIV positive persons receiving ART (CD4+ cell count ≥125 cells/mm^3^ and HIV RNA plasma levels <50 copies/ml) in one of three arms: continuous, 7 days on/7 days off and 5 days on/2 days off treatment. Primary endpoint was ART treatment failure determined by plasma HIV RNA level, CD4+ cell count decrease, death attributed to study participation, or opportunistic infection.

**Results:**

Following enrollment of 32 participants, the 7 days on/7 days off arm was closed because of a failure rate of 31%. Six of 52 (11.5%) participants in the 5 days on/2 days off arm failed. Five had virologic failure and one participant had immunologic failure. Eleven of 51 (21.6%) participants in the continuous treatment arm failed. Nine had virologic failure with 1 death (lactic acidosis) and 1 clinical failure (extra-pulmonary TB). The upper 97.5% confidence boundary for the difference between the percent of non-failures in the 5 days on/2 days off arm (88.5% non-failure) compared to continuous treatment (78.4% non failure) was 4.8% which is well within the preset non-inferiority margin of 15%. No significant difference was found in time to failure in the 2 study arms (p = 0.39).

**Conclusions:**

Short cycle 5 days on/2 days off intermittent ART was at least as effective as continuous therapy.

**Trial Registration:**

ClinicalTrials.gov NCT00339456

## Introduction

Combination antiretroviral therapy (ART) has significantly decreased morbidity and mortality for HIV-infected persons who have access to such therapy.[1]–[3] However, it is now clear that currently available medications will not eradicate or “cure” HIV disease making lifelong therapy a necessity. Although there have been advances in treatment options that have reduced pill burden and frequency of dosing, the need for daily dosing poses challenges for long term adherence. While newer formulations of antiretroviral drugs (ARVs) have reduced certain toxicities, other significant complications remain and continued exposure has revealed new toxicities.[4], [5] Long term toxicities can affect adherence as well as the effectiveness of treatment.[6] Several significant international and domestic initiatives have greatly expanded ART in resource limited settings. Further reductions in the cost of ARVs could increase the reach of such programs. Recently, guidance has shifted to earlier initiation of ART and ART has been suggested as a prevention strategy.[7], [8] ART approaches that reduce cost and toxicity while potentially increasing adherence could significantly enhance the feasibility of the implementation of such strategies on a large scale.

Researchers have previously evaluated several approaches of structured treatment interruptions to enhance adherence and reduce toxicity and cost.[9]–[11] It is now clear that long cycle interruptions of ART that allow a rebound of plasma HIV RNA are not clinically beneficial. The SMART and TRIVICAN trials showed significant increases in death and opportunistic infections with long cycles of treatment interruptions, consistent with previous trials showing virologic and immunologic failure with such strategies.[12]–[14] In sharp contrast, short cycle interruptions are designed specifically to maintain suppression of plasma HIV RNA below the limit of assay detection. In pilot studies conducted in the United States of short cycle interruptions designed to maintain suppression of plasma HIV RNA below the limit of detection, we observed that 7 days on and 7 days off ARV maintained suppression of plasma HIV RNA below the limit of detection and reduced certain laboratory markers of toxicity with certain regimens.[10], [15] However, strict adherence to the 7 day cycles was essential for the success of such an approach. Because of the potential difficulty for certain individuals to maintain strict adherence to such cycles, we reasoned that more “user-friendly” short cycle ARV interruption approaches could have greater implementation feasibility and clinical relevance. Based on these findings, we initiated a randomized, controlled, non-inferiority trial of two short cycle intermittent ART regimens (7 days on and 7 days off and 5 days on and 2 days off) compared to continuous ART. Because of the potential applicability of those strategies to resource limited settings, the study was conducted in Uganda. The 5 days on 2 days off regimen was based on the known kinetics of the rebound of plasma HIV RNA following a discontinuation of ART and the potential clinical applicability of a regimen that allowed treatment interruptions on weekends which could allow for novel approaches such as directly observed therapy (DOT) in certain settings such as schools and the workplace and could be useful in expanding the use of ART earlier in the course of disease.[16]

We here report an evaluation of a randomized, controlled, non-inferiority trial of two regimens of short cycle intermittent compared to continuous ART on virologic, immunologic, toxicity and adherence parameters.

## Methods

The protocol for this trial and supporting CONSORT checklist are available as supporting information; see Checklist S1 and Protocol S1.

### Participants

Ethics Statement: The study protocol was reviewed and approved by the Uganda National Council for Science and Technology (UNCST), and by the Institutional Review Boards (IRBs) of the National AIDS Research Committee of the UNCST, Uganda and the National Institute of Allergy and Infectious Diseases, National Institutes of Health (NIAID/NIH).

HIV-infected persons receiving ART at the Joint Clinical Research Center (JCRC) in Kampala, Uganda, with a CD4+ cell count equal to or greater than 125 cells/mm^3^ of whole blood and plasma HIV RNA below 50 copies per milliliter of plasma were eligible following signed informed consent. A confirmatory HIV test was required prior to enrollment. Participants were required to be receiving at least 3 ARVs including a protease inhibitor or the non-nucleoside reverse transcriptase inhibitor, efavirenz. Patients receiving a nevirapine-based regime were required to switch to a protease inhibitor or efavirenz if they were randomized to the interrupted arm. Nine patients in the 5 days on/2 days off arm and 7 participants in the 7 days on/7 days off arm were on nevirapine and switched to efavirenz for study enrollment. Stavudine was dosed by weight, participants weighing >60 kg received 40 mg, those weighing <60 kg received 30 mg. All participants paid for their ARVs, consistent with standard practice at the JCRC. It should be noted that at the time the study was initiated, large international programs to support the cost of ART had not begun. Such programming was not available until a large number of patients had already been enrolled. It was the decision of the investigators and the review boards that changing the payment practices mid-study could bias the results. Trial oversight was provided by and independent Data Safety and Monitoring Board (DSMB). Clinicaltrials.gov registry number NCT00339456.

### Study design

The study was designed to test non-inferiority of two short cycle intermittent ART regimens (7 days on/7 days off and 5 days on/2 days off) compared to continuous ART with 57 patients (52 plus 5 allowed for attrition) randomized in a 1-1-1 manner to each arm and followed for 72 weeks (73 weeks for the interrupted arms because all lab tests were done during on-treatment periods). The primary endpoint was ART treatment failure determined by a plasma HIV RNA level equal to or greater than 10,000 copies on any one evaluation, a plasma HIV RNA level equal to or greater than 1,000 copies on two consecutive measurements, a plasma HIV RNA level greater than 400 copies/ml at the end of the study, a CD4+ cell count decrease of greater than 30 percent from baseline on 2 consecutive measurements, death attributed to study participation or occurrence of an opportunistic infection. The primary endpoint was changed during the study by the protocol team from <50 copies/ml to <400 copies/ml (end of study) to eliminate the inclusion of low-level viral blips. Patients who experienced treatment failure received standard clinical care in Uganda including switching of ARV regimen, where appropriate. They were followed until 30 days after their plasma viremia and/or CD4+ cell count returned to baseline if they changed therapy or for 30 days from the time of treatment failure if they decided not to change therapy. Laboratory safety monitoring and HIV viral load measurements (Amplicor HIV-1 Monitor v1.5 – Roche, Switzerland ) were performed on site at the JCRC. HIV genotyping (Trugene HIV-1 Genotyping Kit, Visible Genetics – Siemens Healthcare Diagnostics, Inc., Tarrytown, NY) was performed off-site at SAIC-Frederick, Inc., Frederick, Maryland.

### Data collection and follow-up

Participants were evaluated for standard clinical, virologic, immunologic, and adherence parameters at weeks 2 and 4 following enrollment (interrupted arms only, not included in outcome analysis) and every 6 weeks thereafter including toxicity monitoring (renal toxicity, ALT and AST were evaluated every 12 weeks and lipid profiles were done every 6 months). Lipodystropy, peripheral neuropathy and lactic acidosis were determined by the treating physician's assessment, clinical symptoms and laboratory confirmation (serum lactate). All laboratory evaluations were provided to the participants free of charge. Intervening visits were scheduled based on clinical standard of care or relevant failure parameters. All evaluations except the end of study evaluation were performed at the end of the off drug period in the intermittent arm. Adherence to ART was measured using patient maintained study calendars which were recorded at all study visits, adherence rates based on self-reported diaries were then calculated based on the percentage of drugs taken.

### Interim monitoring of safety and efficacy

An independent data and safety monitoring board (DSMB) reviewed all safety data and treatment failures on a bi-annual basis and at least yearly after 50% of participants were enrolled in each treatment arm.

### Statistical analysis

A sample size of 57 individuals in each treatment group (52 plus 5 allowed for attrition) was determined for a 95% non-failure rate in each study group with a non-inferiority margin of 15%, an alpha (type I error) of 0.025 and 81% power using the Farrington and Manning method.[17] The non-inferiority of the 5 days on/2 days off ART arm to continuous ART arm was assessed by constructing the upper limit of a 97.5% confidence interval for the difference in proportions, continuous ART minus 5 days on/2 days off, of those successfully completing the study.[18]

Paired differences and the median correlations, determined by the Spearman rank method, were tested for significance by the Wilcoxon sign rank test. The Wilcoxon two-sample test was used to compare independent groups, and Fisher's exact test was used to compare frequencies of categorical variables. Comparison of Kaplan-Meier survival curves was done by the log-rank test. The significance of the ratios of rates of occurrences of adverse events was determined by the exact rate ratio test based on the Poisson distribution. Exact methods were used so that p values and confidence intervals are properly defined even when the counts are very small or zero.[19] The calculations were done using the ‘rateratio test’ R package. [20] Adjustment of p values for multiple testing was performed by the Bonferroni method.

### Role of the funding source

The study was funded through the Division of Intramural Research, NIAID/NIH. SJR, CWH, MD, RTD, ASF, TCQ & MAD were employees of the NIAID/NIH during the study design and implementation period.

## Results

### Study Population

**Figure 1:**
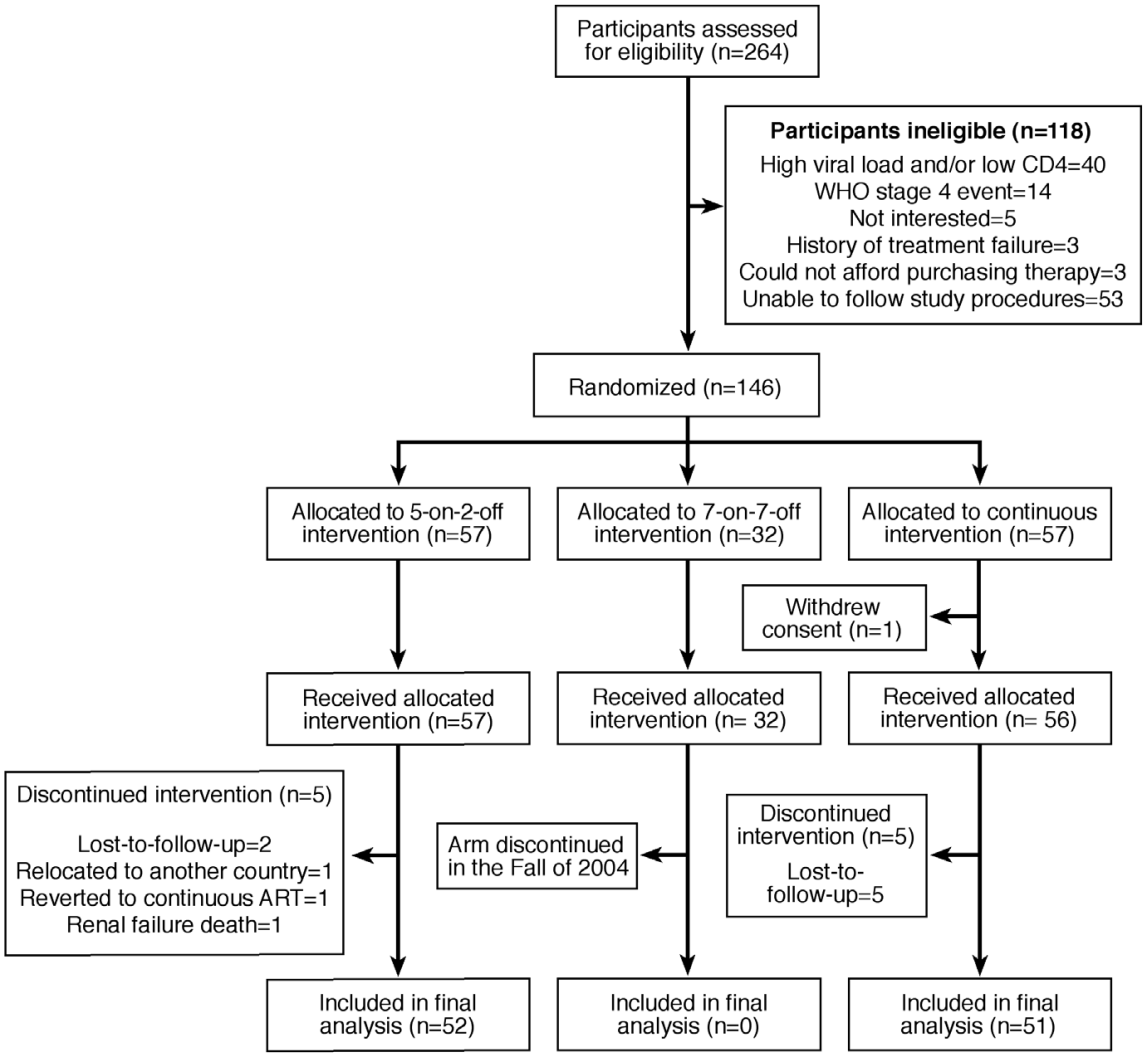
Flow diagram for study eligibility and follow-up.

A total of 146 HIV-positive participants were enrolled in the trial between 2002 and 2005: 32 in the 7 days on/7 days off arm, 57 in the 5 days on/2 days off arm and 57 in the continuous arm. Five individuals in the 5 days on 2 days off group left the study (2 lost-to-follow-up, 1 relocated, 1 reverted to continuous ART and 1 with renal failure died). Five in the continuous arm were lost-to-follow-up resulting in 52 with data in the interrupted and 51 in the continuous arm at week 72/73 (Figure 1). Fifty-six participants in the continuous arm (one withdrew without participating and was excluded from analysis) and 57 participants in the 5 days on/2 days off ART are included in the intent-to-treat analysis. Table 1 summarizes the key baseline characteristics of the participants. The groups were similar in all characteristics upon study entry.

**Table 1:** Baseline Characteristics.

Baseline Characteristic	Continuous n = 56	5 Days On/2 Days Off n = 57	7 Days On/7 Days Off** n = 32
Age (year)	38 (33,44)*	38 (34, 46)	38 (32, 43)
Females	36 (64%)	35 (61%)	21 (66%)
Males	20 (36%)	22 (39%)	11 (34%)
BMI#	25 (22,29)	25 (23, 29)	25 (22, 29)
CD4 T cells (cells/mm)	262 (192,389)	264 (219, 405)	268 (213, 375)
HIV RNA (copies/ml)	<50 (<50, <50)	<50 (<50, <50)	<50 (<50, <50)
PI based regimen	1 (1.8%)	1 (1.8%)	2 (6.2%)
NNRTI based regimen	55 (98%)	56 (98%)	30 (94%)
Stavudine backbone	40 (71%)	44 (77%)	21 (66%)
Zidovudine backbone	16 (29%)	13 (23%)	11 (34%)
Pre-study ART exposure time (weeks)(IQR)	49 (29–83)	48 (30–69)	46 (33,76)

# n = 55 in Continuous arm.

*Median (IQR).

**The 7 days on and 7 days off arm was closed following enrollment of 32 participants because of a 31% failure rate in this group found during interim analysis.

### Primary endpoint

Following the enrollment of 32 participants in the 7 days on and 7 days off arm, the DSMB agreed with the principal investigator to terminate that arm because of a failure rate of 31% found during the interim analysis in the fall of 2004 (3 participants had withdrawn and 9/29 remaining participants had failed).

**Figure 2:**
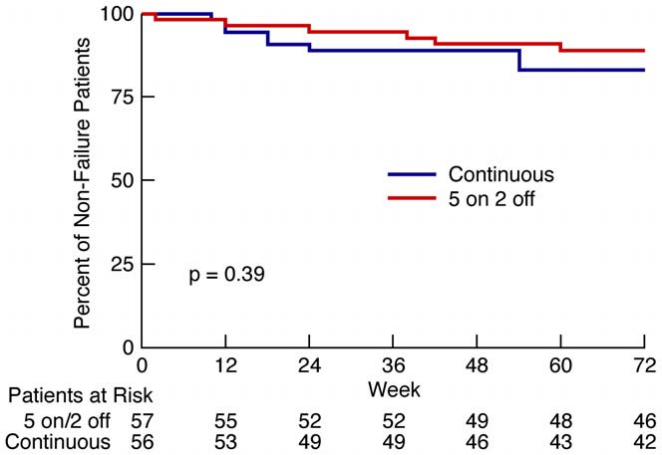
Kaplan-Meier Survival Curve with Time to Failure. No difference was found in the time to failure in the 2 groups during the 72/73 week study.

Six of 52 (11.5%) participants who completed the 5 days on/2 days off arm failed. Five of these participants had virologic failure all with a viral load greater than 10,000 copies/ml (median VL = 50,130 copies/ml; interquartile range (IQR) 14,398 to 50,551 and one participant had immunologic failure. In comparison, eleven of 51 (21.6%) participants who completed the continuous treatment arm failed. Nine of these participants had virologic failure. Six failed with a viral load greater than 10,000 copies/ml, one with a viral load greater than 1,000 and 2 with a viral load greater than 400 at week 72(median VL = 24,410 copies/ml; IQR: 4,394 to 86,316 copies/ml) The remaining 2 failures included 1 death (lactic acidosis) and 1 clinical failure (extra-pulmonary TB). The upper 97.5% confidence boundary for the difference between the percent of non-failures in the 5 days on/2 days off (88.5% non-failure) arm compared to continuous treatment (78.4% non failure) was 4.8% which is well within the preset non-inferiority margin of 15%. In the modified intent-to-treat analysis which designated the 5 in each group who discontinued treatment as failures, the upper 97.5% confidence boundary for the difference between the percent of non-failures in the 5 days on/2 days off, 80.7% (46 of 57), and that in the continuously treated, 71.4% (40 of 56) was 6.6% which is within the preset non-inferiority margin of 15%. In the intent-to-treat analysis with 57 in each group, the upper 97.5% confidence boundary for the difference between the 80.7% (46 of 57) non-failures in the 5 days on/2 days off, and the 70.2% (40 of 57) non-failures in the continuously treated arm was 5.4% which is again well within the preset non-inferiority margin of 15%. No significant difference was found in time to failure in the 2 study arms, p = 0.39 (Figure 2).

### Immunologic responses to treatment

**Figure 3:**
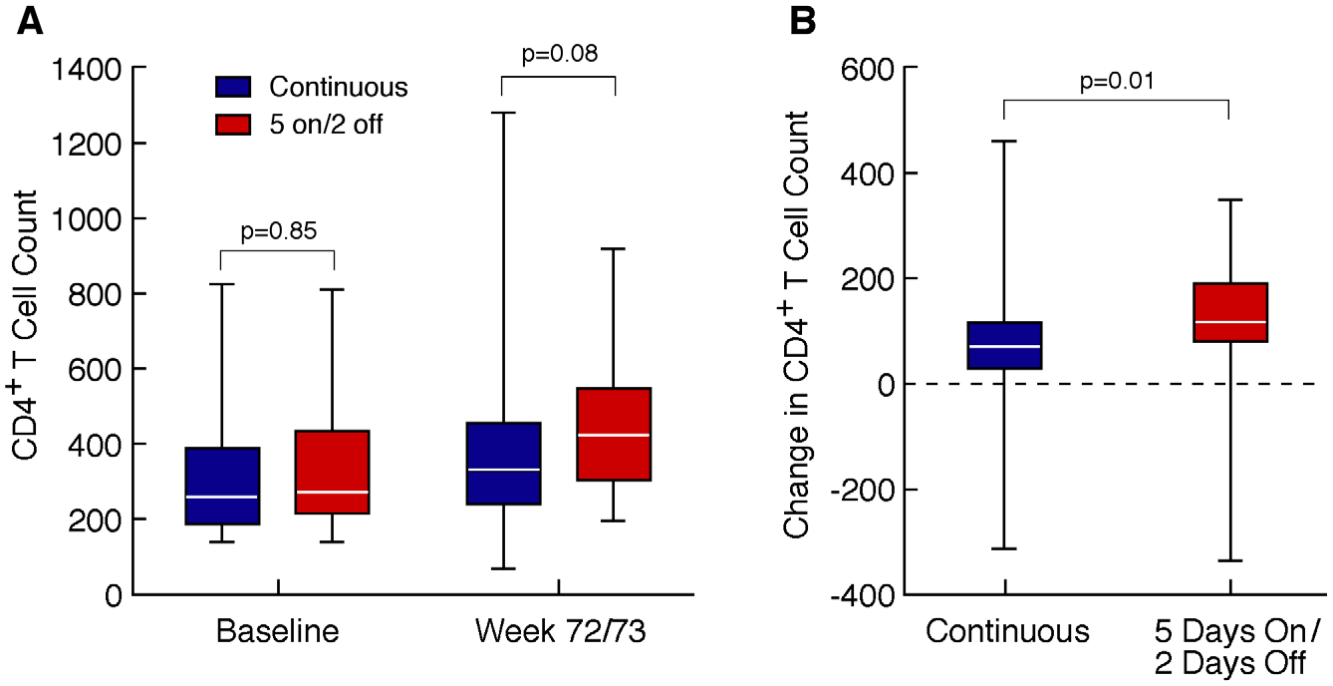
A. Change in CD4+ T cells for participants successfully completing 72/73 weeks. The CD4+ T cell counts in the continuously treated (N = 40) and in the 5 days on/2 days off (N = 46) arms were similar at baseline (255 and 271/mm^3^, respectively, p = 0.85) and marginally different at week 72/73 (330 and 429/mm^3^, respectively, p = 0.08). B. The median paired difference in CD4+ T cells between baseline and week 72/73 was greater in the 5 days on/2 days off group (114/mm^3^) than in the continuously treated group (68/mm^3^). (p = 0.01). The lines across the box indicates the median values; the boxes contain the 25–75% interquartile range; and the whiskers extend to the highest and lowest values.

As mentioned above, only 1 participant in the 5 days on/2 days off arm failed by protocol defined immunologic criteria (CD4 decrease of >30% on 2 consecutive measurements) at week 12 of follow-up. This participant remained virologically suppressed (<50 copies/ml) through week 73 suggesting that this was not a true failure but merely a fluctuation in CD4+ T cell count. CD4+ T cell counts were correlated with weeks on treatment in the continuous treatment arm (median r = 0.54, p<0.001) and in the 5 days on, 2 days off arm (r = 0.57, p<0.001). The correlations in the two groups were not statistically different (p = 0.33). For the 46 individuals in the 5 days on/2 off arm and the 40 in the continuously treated arm who successfully completed the study the median CD4+ T cell counts at baseline were similar in the two arms (255 and 271/mm^3^, respectively, p = 0.85), at week 24 (347 and 293/mm^3^, respectively, p = 0.30), at week 48 (399 and 318/mm^3^, respectively, p = 0.55) and marginally different at week 72/73 (p = 0.08)(Figure 3A). The median paired changes in CD4+ T cells from baseline were not statistically different at week 24 (48 and 15/mm^3^, respectively, p = 0.21) nor at week 48 (90 and 69/mm^3^, respectively, p = 0.90). The median paired difference in CD4+ T cells was statistically greater in the 5 days on/2 days off arm at week 72/73 (p = 0.01)(Figure 3B) None of the virologic failures experienced a corresponding immunologic failure during follow-up.

### Toxicity

The rates of lactic acidosis, lipodystrophy and peripheral neuropathy were compared among participants receiving stavudine within the continuous versus 5 days on/2 days off ART groups. The data were normalized for comparison between groups by reporting data as events per 100 person years. Among the 40 participants receiving continuous stavudine, 5 cases of lactic acidosis as determined by clinical findings and arterial blood levels of lactate occurred (resulting in one death), with an incidence of 11.4/100 person years, whereas no occurrences of lactic acidosis occurred among 44 participants receiving intermittent ART that included stavudine (p = 0.04). A reduction in lipodystrophy was also observed with 5 cases in the continuous treatment arm (incidence 11.4/100 person-years) compared with 1 case in the intermittent treatment arm (incidence 1.8/100 person-years) (p = 0.13). The rate of lipodystrophy in the continuous arm was 6.2 times the rate in the 5 days on and 2 days off arm (95% confidence interval {CI}: 0.7, 293). The rates of peripheral neuropathy were similar in both arms (Table 2) with the occurrence in the continuous arm 1.5 times (95% CI: 0.4, 6.2) that in the intermittent treatment arm. Because there were no cases of lactic acidosis in the 5 days on 2 days off arm, the ratio comparing the continuous and intermittent rates was infinite with a 95% CI lower limit of 1.13, i.e. greater than 1. Participants who developed lipodystrophy, peripheral neuropathy or lactic acidosis were switched from stavudine to zidovudine as standard of care at the time.

**Table 2:** Toxicity.

Event (cases/100py)*	Continuous	5 Days On/2 Days Off	p-value
Lactic Acidosis	11.4	0	0.04
Lipodystrophy	11.4	1.8	0.13
Peripheral neuropathy	13.7	9.2	0.72
Cholesterol/LFTs			
Creatinine**			
at week 72/73			
Median (IQR) [N]			
Total Cholesterol (mg/dL)	176 (141-194) [38]	192 (174-220) [43]	0.15
LDL Cholesterol (mg/dL)	96 (76-127) [36]	123 (106-139) [43]	0.08
HDL Cholesterol (mg/dL)	42 (35-50) [37]	45 (34-58) [43]	0.99
Triglycerides (mg/dL)	149 (88-212) [36]	144 (98-212) [42]	0.99
AST (IU/L)	21 (16-31) [27]	22 (17-24) [35]	0.99
ALT (IU/L)	20 (14-32) [27]	18 (13-25) [35]	0.99
Creatinine (mg/dL)	0.74 (0.62-0.83) [35]	0.76 (0.68-090) [43]	0.54

*stavudine patient years, subanalysis of participants receiving stavudine containing regimens.

**Cholesterol, liver function tests and creatinine for patients followed 72/73 weeks (all HAART regimens).

One patient receiving 5 days on and 2 days off ART died from chronic renal failure that predated enrollment; the event was determined to be unrelated to the study by investigators in Uganda. The patient had a plasma HIV RNA level of less than 50 copies per milliliter of plasma at death and a CD4+ T cell count at baseline and death of 171 and 349, respectively. No other significant hepatotoxicity, renal toxicity or anemia were observed during follow up with similar AST, ALT and creatinine measurements between study arms at 73 weeks (Table 2). Median LDL cholesterol levels were slightly lower at 73 weeks comparing continuous versus intermittent treatment arms (96 versus 123, p = 0.08).

### Adherence

Overall adherence to ARVs for the study was greater than 95% in both study arms based on participant diaries. Pharmacy records were consistent with the high level of reported adherence measured during the study (data not shown).

### Antiretroviral resistance

Clinical monitoring of mutations associated with antiretroviral drugs resistance was not available in Uganda during the study period. Consistent with standard clinical care at the JCRC and per protocol, and with review and monitoring by the DSMB and the IRB, patients were evaluated for failure by laboratory and clinical evaluations and treated according to standard clinical practice including switching of ARV regimen where appropriate. Genotypic evaluation of mutations associated with ARV resistance was performed by SAIC-Frederick, Inc., Frederick, Maryland and the results were made available to clinicians in Uganda.

Genotyping was performed on 4 out of 5 of the participants in the 5 days on 2 days off arm at the first viral load measured >1000 copies/ml. Each participant was receiving an efavirenz-based regimen that included lamivudine and each had mutations consistent with resistance to efavirenz and lamivudine (Table 3). No participant developed thymidine analogue mutations (TAMS) or evidence of nucleoside analogue resistance apart from one individual who developed an A62V insertion as previously reported.[21] Genotyping was also performed on 5 of the 7 participants in the continuous treatment arm who developed virologic failure during follow-up (first VL>1000 copies/ml). Two participants had wild type virus, 3 had mutations consistent with resistance to efavirenz and one had a mutation consistent with resistance to lamivudine. None of the participants in the continuous arm had TAMS or nucleoside analogue resistance, as previously reported.[21]

**Table 3:** Reverse Transcriptase Genotypic Resistance Among Participants with Virologic Failure.

Participant	Study Arm	Failure Week	HIV RNA Level (copies/ml)	Major RT Mutations
6	5 on/2 off	38	50 130	K103N, M184V
31	5 on/2 off	42	60 899	K103N, M184V
248	5 on/2 off	12	14 398	K101P, K103N
				M184V
258	5 on/2 off	60	50 551	K103N, V1081
				M184V, P225H
9	Continuous	18	24 400	K103N
21	Continuous	54	205 517	None
81	Continuous	72	4 394	Genotype failed
98	Continuous	24	56 800	K103N, M1841
245	Continuous	18	18 933	None
256	Continuous	54	128 231	K103N

## Discussion

The present study demonstrates that short cycle intermittent ART defined as 5 days on/2 days off is at least as effective as continuous ART over a 72/73 week follow-up period. The data are consistent with the results of a pilot study that was begun in the United States after this randomized, controlled trial was underway and with a recent report of significantly increased risk of failure with interruptions beyond 2 days.[22], [23] Our findings are also consistent with those of the FOTO study which found that participants on a 5 day on/2 day off interruption strategy receiving tenofovir, emtricitabine and efavirenz maintained virologic suppression at 24 weeks.[24] The primary endpoint, ART treatment failure, was reached by a lower percentage of participants in the intermittent arm than the continuous treatment arm by 73 weeks of study follow-up. The early termination of the 7 days on/7 days off arm due to a high failure rate was consistent with another trial reported after the present study was begun.[25] It is important to point out that failures that have been reported in treatment interruption studies have invariably been associated with a rebound in plasma viremia during the period of intermittent discontinuation of ARVs.[12], [13] Such rebounds are generally associated with the emergence of drug resistance and the depletion of CD4+ T cells.[12], [26] We chose the 5 days on/2 days off regimen of treatment interruption to study because previous experience with the kinetics of plasma rebound following interruption of ART strongly indicates that plasma viremia that had been suppressed below the level of detection (<50 copies per ml) by ART does not generally rebound during the 2 day drug-interruption period.

Although the trial was not powered to show superiority, it was interesting that there were nearly twice as many failures in the continuous arm compared to the 5 days on/2 days off arm (11 and 6, respectively). The adherence data collected indicated high levels of adherence in each arm. However, 2 of 5 participants with virologic failure in the continuous arm evaluated for drug resistance had wild-type virus, suggesting poor adherence. Those results are compatible with the possibility that scheduled interruptions promote adherence during the on-drug period. All participants in the 5 days on/2 days off arm developed genetic mutations consistent with resistance to both lamivudine and efavirenz while 3 of 5 participants in the continuous arm developed mutations consistent with resistance to efavirenz and only 1 developed resistance to lamivudine with a second participant developing lamivudine resistance at a later follow-up visit. Our study was not powered to specifically look at differences in genotypic resistance patterns and also used self-reported adherence, known to be an imperfect measure, limiting our ability to draw any firm conclusions from these findings.[27]

There was evidence of reduced toxicities associated with intermittent ART. This was particularly striking among the subset of participants receiving stavudine containing ART which is commonly used in resource limited settings despite efforts to replace it with other nucleoside reverse transcriptase inhibitors. We observed a marked reduction in the rate of lactic acidosis among participants receiving intermittent stavudine with no cases detected in this group compared to 5 cases in the continuous treatment arm. A similar although not statistically significant reduction in lipodystrophy was also observed among participants receiving intermittent stavudine in our study. This finding is consistent with reports that mitochondrial toxicity associated with stavudine appears to be at least partially dose dependent.[28] Of note, lipodystrophy was physician defined which could result in measurement bias. If larger clinical trials validate reductions in debilitating and fatal adverse reactions observed in this trial, short cycle structured treatment interruptions could have particular relevance for earlier initiation of ART and ART as a viable prevention strategy.

Immunologic responses to ART were robust in both intermittent and continuous treatment arms. Although there was a significantly greater increase in CD4+ T cell counts between arms for patients followed more than 52 weeks, the clinical relevance of this finding is unclear. Larger trials would be needed to validate those results and to determine their clinical significance. Consistent with recent publications from other resource limited settings, immunologic failure was not observed despite several episodes of virologic failure.[29]–[31] Of note, none of the participants in this study who had developed virologic failure and genetic mutations associated with ARV resistance would have been detected by the current WHO guidelines for determining ARV failure and changing to second line regimens based on immunologic criteria alone, highlighting the role of virologic evaluations to determine treatment failure.[32]

The present study has several limitations. It was not powered to evaluate superiority or long term outcomes including death. The protocol included only subjects on efavirenz in the intermittent arms due to the concern of nevirapine resistance. Because nevirapine is extensively used in low and middle income countries this might limit the applicability of our findings. The continuous treatment arm had a higher than expected failure rate than used in the original statistical design which could impact on the overall conclusion of non-inferiority. The methodology used to measure adherence relied on participant diaries which may not reflect the true adherence levels of the different treatment arms in the study. Finally, the patient population at the JCRC tended to be of higher socioeconomic status during the time of the study and thus, most were able to pay for their ART. A larger trial will be needed to more fully address short cycle intermittent therapy among patients representing a wide range of demographies.

Since 5 days on/2 days off intermittent therapy has been shown to be as least as effective as continuous therapy, this strategy could have particular importance for programs of directly observed therapy (DOT) for difficult to treat populations including children and adolescents attending school 5 days a week. It could also be relevant for workplace programs. The possibility of decreased toxicity could be relevant as treatment guidelines shift to initiating ART earlier in the course of disease. Drug treatment costs could also be 29 percent lower with a 5 days on/2 days off treatment regimen. Finally, as guidance shifts to earlier initiation of ART, a less expensive, “user-friendly” approach with reduced toxicity could be significant for scale-up of those strategies, particularly in the developing world.

## Supporting Information

Protocol S1 Trial Protocol.
(0.08 MB PDF)

Checklist S1 CONSORT Checklist.
(0.19 MB DOC)
