# Supplementary material for: A Randomized, Controlled, Trial of Short Cycle Intermittent Compared to Continuous Antiretroviral Therapy for the Treatment of HIV Infection in Uganda
Source: PLoS One. 2010 Apr 22;5(4):e10307. doi: 10.1371/journal.pone.0010307 (PMC2860845; doi:10.1371/journal.pone.0010307)
Supplement: Protocol S1 — Trial Protocol. (0.08 MB PDF) [file pone.0010307.s001.pdf]

TITLE: “A randomized, controlled trial of short cycle intermittent versus continuous HAART for the treatment of chronic HIV infection in Uganda"

Principal investigators: Tom Quinn, NIAID Peter Mugyenyi, MBChB, FRCP Director, Joint Clinical Research Center (JCRC) Kampala, Uganda

Associate investigators: Anthony Fauci, MAID Richard Davey, NIAID Steve Reynolds, NIAID-Uganda

Cissy Kityo Mutuluuza, MBChB, MSc Research Coordinator, JCRC Kampala, Uganda

Roy Mugerwa, MBChB, M.Med
Senior Consultant Physician and PI Uganda-CWRU Research Collaboration

Edward Mbidde, MBChB, M.Med Senior Consultant Physician/Director Uganda Cancer Institute

Version: 5.8, 19 December 2008

Precis

Although highly active antiretroviral therapy (HAART) has been successful in suppressing plasma HIV RNA levels and providing significant clinical benefit in infected patients, it does not eradicate HIV infection. It is now clear that virus replication persists despite undetectable plasma viremia in individuals receiving HAART. In this regard, withdrawing HAART, even after prolonged periods of virus suppression, leads almost invariably to a rapid rebound of plasma viremia. It is also now clear that prolonged, continuous HAART carries a risk of significant toxicity and side effects. In addition, the monetary cost of HAART is prohibitive for many individuals and countries. In terms of cost, 95% of the HIV-infected individuals in the world are beyond the reach of therapy as a direct consequence of the cost of therapy. These observations may argue for a different approach to HAART with the goals of: 1) durable suppression of virus replication, without an attempt at eradication, 2) minimization of toxicity and side effects and improvement in patient life-style, and 3) a reduction in cost Preliminary data from a pilot study conducted at the National Institutes of Allergy and Infectious Diseases, USA, have demonstrated that short cycle structured intermittent therapy, 7 days HAART drug followed by 7 days off HAART has maintained suppression of plasma HIV RNA while preserving CD4+ T cell counts for up to 80 weeks. In addition, there was no evidence for increased HIV in reservoir sites; nor was there evidence for the development of resistance to antiretroviral drugs. Finally, there was a decrease in parameters of toxicity. This approach may have particular applicability for the treatment of HIV in the Southern Hemisphere. Therefore, we propose to study the virologic and immunologic effects of short cycle intermittent versus continuous HAART in HIV-infected individuals from the JCRC (Kampala, Uganda) in a randomized, controlled, intent-to-treat trial. We shall evaluate both the 7 days on-HAART/ 7 days off-HAART as well as a 2 days off HAART/ 5 days on-HAART approach. In December, 2004, the 7/7 arm was discontinued.

Introduction

The use of highly active anti-retroviral therapy (HAART) has resulted in a considerable improvement in mortality and a decrease in adverse events for HIV-infected individuals in the Northern Hemisphere (1). However, eradication of virus in chronically infected individuals has not been attainable. Recently, several investigators have demonstrated that virus replication persists despite prolonged periods of undetectable plasma HIV RNA (2-4). Furthermore, when HAART is discontinued after an extended duration of undetectable plasma viremia, there is a nearly universal and rapid rebound of virus replication as indicated by measurements of plasma HIV RNA (5-8). Even in individuals in whom infectious HIV could not be detected in known reservoirs prior to interruption of HAART, plasma viremia occurred rapidly after withdrawing therapy (5). These data suggest that the currently available therapies will not eradicate HIV in individuals who begin therapy after the acute phase of infection has passed. This disheartening news is compounded by the fact that the antiretroviral drugs approved for the treatment of HIV carry substantial toxicity, particularly when used in combination and for extended periods of time (9-11). Protease inhibitors may have the worst toxicity profile, including renal stones or sludging, hypercholesterolemia, hyperlipidemia, hypertriglyceridemia, hyperglycemia, cardiac dysfunction and lipodystrophy (9-11). However, other agents also have toxicity, and some antiretrovirals, e.g. the non-nucleoside reverse transcriptase inhibitors, may not have been in use long enough to appreciate their long-term effects. Furthermore, adherence can be a significant problem. In certain studies, fewer than 50% of patients were able to take their medications as instructed (12-16). Finally, the monetary cost of

medications and monitoring is prohibitive for many countries and individuals.. Thus, the vast majority of individuals infected with HIV, particularly in Africa and Asia, do not have access to HAART. The devastating consequences of this lack of therapy can be seen in countries where gains in life expectancy over the last few decades have been lost. It is currently estimated that the life expectancy in southern Africa, which had risen from 44 to 59 years from the 1950s to the 1980s, will return to 45 years over the coming decades as a consequences of HIV/AIDS (17). Recently there has been a significant international effort to obtain HAART for the economically underdeveloped areas of the world at reduced prices. Additional mechanisms to reduce the cost of therapy could have a critical impact on the number of individuals with access to HAART.

Recently, through MAID, NIH, USA protocol 00-1-0020, we have demonstrated that short cycles of 7 days off HAART followed by 7 days on HAART can maintain suppression of HIV in plasma and in important reservoir sites such as resting CD4+ T cells and lymph nodes while preserving CD4+ T cell counts for up to 1 year in patients studied in the United States (18). These results are encouraging and indicate that it may be possible to safely reduce the cost of HAART by 50%. In addition, such a regimen may slow the onset of accumulated toxicity while potentially enhancing adherence. In this regard, over a 24-52 week period a significant decrease in serum cholesterol, LDL cholesterol and triglyceride levels was observed (18). However, this was a small pilot study of only 10 individuals who, in general, had < 50 copies/ml of plasma HIV RNA for > 1 year and a mean CD4+ T cell count of 940 cells/mm^3. It is important to evaluate this approach further to determine if it may have more general clinical applicability. In the United States, a randomized, controlled trial of 7 days on HAART followed by 7 days off HAART versus continuous HAART is underway. However, as a consequence of the potential for cost reduction, and hence wider accessibility of treatment, this approach may have particular relevance for individuals in the Southern Hemisphere. For example, as a result of the decrease in the price of HAART in Kampala in 2000 from approximately USD 400/month to USD 50/month, the JCRC has seen an increase in the number of individuals who are receiving HAART there from approximately 1,000 to 3,000. It is estimated that if the cost could be cut in half again, the number of patients that could afford therapy may increase $\geq$ 6-fold, and the majority of the teacher class could afford therapy (P. Mugyenyi, personal communication). In addition, as the Global Fund becomes available, and as countries such as Botswana and Nigeria begin implementation of national antiretroviral drug availability programs, it may be possible to treat considerably more individuals if the cost of drugs were reduced given the limitations on funding for therapy.

In the Southern Hemisphere, a limited number of individuals are able to afford medications, and when they can it is usually during more advanced disease (P. Mugyenyi, personal communication). In order to enroll a broader spectrum of HIV-infected individuals and to more completely evaluate the clinical utility of short cycle intermittent HAART in Kampala, patients on study are required to have a current CD4+ T cell count of $\geq$ 125 cells/mm^3 at enrollment. Although this is lower than the enrollment criteria for the pilot study in the US (300 cells/mm^3), since we did not observe a significant decline in CD4+ T cell counts in the pilot study, and since we exclude patients with a history of significant HIV-related opportunistic infections in this study, the risks to the patients would seem to be minimized. According to withdrawal criteria, patients in whom the CD4+ T cell count drops to 100 cells/mm^3 will be removed from study. There are no data to suggest that there is an increased risk of opportunistic infections with 100 versus 125 CD4+ T cells/mm^3. In addition, all individuals with low CD4+ T cell counts are required to be receiving appropriate prophylaxis against serious opportunistic infections according to existing policies in Uganda.

Treatment in Africa is complicated by the use of generic compounds that do not uniformly undergo

internationally validated quality assurance. Approximately 1/2 of the patients at JCRC receive one or more generic drug in their regimen. However, recently there have been reductions in the price of brand drugs making the latter more competitive, e.g d4T brand costs less than the generic. Also, at JCRC PIs and efavirenz are not available as generic drugs. In order to avoid potential biases based on generic compounds, there will be a randomization for individuals who receive any drug that is generic in their regimen. We shall also record the number of drugs in each regimen that are generic. Although the study is not powered to evaluate the difference between generic and brand drugs, the study may provide insights into potential differences with intermittent therapy.

Since an approach using 7 days on HAART followed by 7 days off HAART has not been evaluated in a community setting, it is possible that the cyclic nature of the therapy may prove difficult with regard to adherence. Thus, an arm of 3 days off HAART followed by 4 days on HAART has been included. This approach may have several advantages in a community setting. Unlike the 7 days approach, individuals would always take or not take their medications the same days of the week. Such a strategy is already utilized at the Joint Clinical Research Center (JCRC) for *Pneumocystis carinii* pneumonia (PCP) prophylaxis (P. Mugyenyi, personal communication). In addition, the durability of the effectiveness of this approach may be optimal given the relatively short off HAART periods. Finally, in patients with lower CD4+ T cell counts, a shorter duration off HAART may have a higher safety profile. A pilot study of this strategy is being evaluated through the NIH protocol mentioned above. In addition, patients participating in a pilot study of 4 days off HAART followed by 3 days on HAART have maintained suppression of HIV plasma viremia for up to 3 months. Again, although the minimum CD4+ T cell count for enrollment was higher than in the present study (300 cells/mm^3), as noted above, this study provides several safe-guards for patients who would be enrolling with lower CD4+ T cell counts. Thus, this approach would not seem to pose any foreseeable risks beyond those of the 7 days approach.

There are several ethical issues that arise when HIV therapy protocols are considered in the Southern Hemisphere. One relates to continuing access to therapy once the study has ended. For this study, antiretroviral or other HIV-therapy is not dependent on the study protocol; all participants are currently receiving HAART through their own means. Thus, it would be expected that this could be maintained in the majority of these individuals beyond study participation. There may also be objections regarding the `experimentation' of therapeutic approaches that would not be contemplated in the Northern Hemisphere. Since all aspects of this study are being performed in the United States, this is not an issue for the present study. Finally, this strategy has direct applicability to individuals living with HIV in the Southern Hemisphere.

In this randomized, controlled, intent-to-treat trial we will evaluate the virologic equivalence of short cycle intermittent and continuous HAART. The results of this study could have a significant impact on the global effort to treat HIV.

Objective

To evaluate the effects of short cycle intermittent versus continuous HAART on viral load

Secondary objectives

1. change in CD4+ T-cell counts change in
2. virus resistance patterns Toxicity and side
3. effects Quality of life Adherence

Study design

This intent-to-treat, randomized, controlled trial of continuous versus 2 approaches to short cycle intermittent HAART will consist of 171 patients receiving HAART with a current CD4+ T cell count > 125 cells/mm^3 and a plasma HIV RNA level < 500 copies/ml > 3 months prior to screening and < 50 copies/ml on 1 screening value. December 2004, the 7/7 arm was discontinued. The study continues with the continuous therapy and 5/2 therapy arms. Although only 1 screening visit is required, if a patient's laboratory values are within the range of the assay's variability for inclusion and exclusion criteria, or if the results suggest the possibility of laboratory error, there may be a second evaluation; criteria must be met on only 1 determination. Patients with a CD4 count at day 0 less <125 cells/mm3 or a plasma HIV RNA level >500 copies/ml at day 0 will not continue in the study, will be switched to continuous therapy if they were randomized to an intermittent arm, will be returned to their primary physician and will be replaced on a one-to-one basis. These patients will not be followed by the study. The study will be conducted at the JCRC in Kampala, Uganda after a Federal-wide Assurance has been obtained. Fifty-seven individuals will be randomized to receive 7 days on HAART followed by 7 days off HAART for 72 weeks or until failure; fifty-seven patients will be randomized to receive 5 days on HAART followed by 2 days off HAART; fifty-seven patients will be randomized to continuous HAART. Patients must be receiving at least 3 antiretroviral agents including either a protease inhibitor (PI), abacavir or a non-nucleoside reverse transcriptase inhibitor (NNRTI) for 90 days prior to enrollment and they must be receiving a PI-containing or efavirenz-containing regimen at enrollment. If an individual was originally receiving a non-study drug, e.g. nevirapine, they must switch to a PI or efavirenz at least 30 days prior to enrollment and have a viral load < 50 copies/ml after being on the new regimen for at least 14 days prior to, enrollment. If patients are receiving abacavir as part of a triple NRTI regimen, they must switch to a PI or efavirenz at least 30 days prior to enrollment and have a viral load < 50 copies/ml after being on the new regimen for at least 14 days prior to enrollment. If patients are receiving abacavir as an NRTI component of a PI- or efavirenz-containing regimen, they must switch to a different NRTI at least 30 days prior to enrollment and have a viral load < 50 copies/ml after being on the new regimen for at least 14 days prior to enrollment. Patients who are randomized to receive continuous therapy allowed to take nevirapine after enrollment. Patients cannot be receiving ddl-Tenofovir regimens. Individuals must not be receiving salvage therapy or have clinical evidence of resistance to antiretroviral agents prior to enrollment.

The primary endpoint shall be the number of individuals in each cohort with a plasma HIV RNA < 50 copies/ml at 72 weeks. Secondary endpoints shall include lymphocyte subset evaluations, the number of, and reason for, individuals changing their antiretroviral therapy and laboratory evaluations of toxicity including triglycerides, amylase, cholesterol and markers of liver function. Other secondary end points will include Quality of life and adherence. Patients will undergo evaluations at least every 6 weeks including laboratory measurements for the duration of the study. In addition, for patients randomized to an intermittent arm there will be an evaluation at weeks 2 and 4 of study participation and there will be a blood draw for viral load and CD4+ T cell count week 73 after a final on-HAART period. The primary endpoint for statistical purposes will be the final on-HAART period for the intermittent group.

Failure will be determined as plasma HIV RNA > 1000 < 10,000 copies/ml and/or a decrease in

CD4+ T cells of > 30% of baseline or and absolute CD4+ T cell count < 100 cells/mm^3 on 2 consecutive measurements. If an initial failure criterion is met, patients will have a repeat evaluation in 2 weeks. A single, confirmed HIV RNA of > 10,000 will constitute an independent failure criterion. Individuals randomized to receive intermittent HAART who fail will be changed to continuous HAART and monitored for 30 days after their plasma viremia and/or CD4+ T cell count returns to baseline, depending on the failure criteria achieved. Failure criteria will also be met if an individual develops an opportunistic infection. Although the development of an 01 may not be related to the study, for safety purposes that will be considered a definitive failure regardless of patient arm. If this occurs that patient will be followed for 30 days after the resolution of the opportunistic infection. Individuals who fail continuous HAART will be treated according to standard care for changing therapy and will be followed monthly until 30 days after their plasma viremia and/or CD4+ T cell count return to baseline if they change therapy or for 30 days from failure if they decide not to change therapy. Genotypic and phenotypic resistance testing may be done to facilitate the care of patients who fail a treatment arm.

Randomization and Blinding

Patients will be randomized through JCRC in a 1-1-1 manner. There will be 57 patients per arm. There will be a sub-randomization based on CD4+ T cell counts > or < 200 cells/mm^3 and a separate sub-randomization based on whether patients are receiving any generic drug in their regimen or all brand antiretroviral drugs. This is an open-label study without blinding.

Patient Selection and Criteria

<u>Inclusion Criteria</u>

1. Documentation of HIV-1 infection by licensed ELISA test kit and confirmed by a second method (e.g. Western Blot).
2. Absolute CD4+ T-cell count of > 125/mm^3 within 30 days before randomization (For patients who are status post-splenectomy, also CD4+ T-cell >20%).
3. If the CD4+ T cell count is < 200 cells/mm^3, the patient must be receiving PCP prophylaxis.
4. Receiving at least 3-drug HAART with the most recent viral load test prior to screening <500 copies/ml. Patients must be receiving 3 drug HAART containing an NNRTI, abacavir or PI for at least 90 days prior to enrollment and at least 1 PI or efavirenz for 30 days prior to enrollment.
5. A viral load of <50 copies/ml prior to enrollment.
6. Age at least 18 years and above.
7. For women of childbearing potential, a negative pregnancy test (serum or urine) is required within 14 days prior to randomization.
8. Laboratory values (within 30 days prior to randomization):
    a. AST no more than 5 X the upper limit of normal (ULN).
    b. Total or direct bilirubin no more than 2 X ULN unless there is a pattern consistent with Gilbert's syndrome or the patient is receiving indinavir.
    c. Creatinine no more than 2.0 mg/dL.
    d. Platelet count at least 50,000/µl.
9. Written consent to participate in the trial
10. Patient financially capable of purchasing the drugs uninterrupted for at least 72 weeks (the duration of the study).

Exclusion Criteria

1. Concurrent malignancy, or any other disease state, requiring cytotoxic chemotherapy.
2. Symptomatic for significant HIV-related illnesses, such as opportunistic infections and malignancies other than mucocutaneous Kaposi's sarcoma. A history of AIDS defining opportunistic infections other than mucocutaneous Kaposi's sarcoma or candida or treated tuberculosis.
3. Use of experimental antiretrovirals < 6 months prior to enrollment. An exception may be made for hydroxeurea according to the judgment of the Principal Investigator.
4. Pregnancy or breastfeeding.
5. Significant cardiac, pulmonary, kidney, rheumatologic, gastrointestinal, or CNS disease as detectable on routine history, physical examination, or screening laboratory studies. If an abnormality is a contraindication to a specific drug, an alternative drug within the same class may be used, as outlined below.
6. Psychiatric illness that, in the opinion of the PI, might interfere with study compliance.
7. Active substance abuse or history of prior substance abuse that may interfere with protocol compliance or compromise patient safety.
8. Refusal to practice safe sex or use precautions against pregnancy (effective birth control with barrier contraceptives or abstinence).
9. Known history or laboratory evidence of chronic hepatitis B infection including surface antigen positivity.
10. Receiving salvage HAART, i.e. evidence of clinical resistance to licensed antiretrovirals as indicated by clinical progression, an elevated viral load or declining CD4+ T cell count while receiving antiretroviral therapy, or receiving sub-optimal antiretroviral therapy prior to HAART.
11. Patients currently receiving nevirapine or abacavir are excluded.

Patients who become pregnant or begin breastfeeding during participation will be withdrawn from the study. Individuals accepted into the protocol who subsequently violate exclusion criteria 6-8 will be offered further counseling and psychiatric evaluation if indicated. If the situation is not resolved within a reasonable period of time, the PI, in consultation with the care team, may terminate subject participation.

Schedule of visits and procedures

The frequency of monitoring is outlined below, but may be modified on an individual basis. Potential candidates will have at least 1 screening visit within 30 days of enrollment to determine eligibility according tot he criteria mentioned above. Individuals randomized to receive continuous or intermittent HAART will have clinical and laboratory evaluations at least every 6 weeks with viral load, FACS analysis for the duration of the study. Safety labs will be performed every six months and at the final visit for patients receiving intermittent therapy; however, creatinine levels will be checked every other visit (every 12 weeks). In addition, all patients will have an evaluation at weeks 2 and 4 of study participation. If a patient meets a viral load or CD4+ T cell count criteria for failure, they will have an additional visit in 2 weeks to evaluate if consecutive failure criteria are met. For patients on the intermittent arm, all scheduled evaluations will be made after the off-drug period. For the intermittent arms, an additional final measurement will be made after a final 7 or 5 days on-drug period. In addition,

patients on the intermittent arm will be required to have an evaluation after week 2 and week 4 and week 73 after a final on-HAART period.

Adherence, a secondary objective, will be measured by 1) inquiries regarding adherence that is recorded in the study record, 2) patient calendars and 3) pharmacy logs. This information will be collected and evaluated at the conclusion of the study and patients will be rated according to the percent of drugs they took: >90%, 80-90%, 70-80% and <70%. The clinical failure rates will be compared to adherence rates as has been described in previously reported studies.

Screening and enrollment

1. Within 30 days prior to randomization:
    a. A baseline history, clinical evaluation and recording of medications according to standard procedures
    b. Absolute $CD4^+$ T cell count. (For participants who are status post splenectomy, also obtain a $CD4^+$ T cell % of lymphocytes.)
    c. Hematology panel (complete blood count with differential and platelet count), chemistry panel (including total cholesterol with subsets, triglycerides, amylase, AST, total bilirubin, and creatinine).
    d. Plasma HIV RNA level
2. Within 14 days before randomization:
   Women of childbearing potential must have a serum or urine pregnancy test.
3. Documentation of the lowest recorded CD4+ T-cell count, the baseline viral load, and the viral load after HAART with associated dates.
4. Hepatitis serology

Routinely scheduled visits

Targeted history and physical examination, including evaluation of life-style issues and side effects
Plasma HIV RNA level (routine samples performed at JCRC; plasma will be cryopreserved for additional measurements at NIH)
Review of concomitant treatments
FACS profile including absolute CD4+ T-cell count (performed at JCRC)
Adherence

Weeks 2 and 4 and last on-drug period visits (intermittent HAART)

Plasma HIV RNA level

Every 6 months

Routine safety labs (performed at JCRC) including fasting lipid panel, liver enzymes, CBC, UA, chemistry panel.)

Every other visit (12 weeks)

Creatinine levels

Study medications

HAART medications

Participants must be receiving at least 3 licensed antiretroviral medications including either an

NNRTI or a PI for 90 days prior to enrollment and a PI or efavirenz for 30 days prior to enrollment. Patients will continue their current drug regimen after enrollment regardless of the arm to which they are randomized. Patients who are randomized to receive continuous therapy allowed to take nevirapine or abacavir after enrollment. Adherence will be assessed by the study co-ordinator and/or case manager through questioning at each visit. Adherence assessments will be recorded with each visit. Quality of life will be assessed through an abbreviated form based on the Wu analysis.

Other medications

Patients must report all long-term medications, including traditional or herbal, taken while enrolled during this study. Patients may not take other approved or experimental antiretroviral agents during the course of their participation in the study without the permission of the PI or designate. Patients will be monitored for the necessity of opportunistic infection prophylaxis and interactions with anti-tuberculosis drugs in keeping with standard clinical practice. According to standard clinical practice in Uganda, primary prophylaxis for cryptococcal disease is not provided. However, secondary prophylaxis is standard clinical care.

Risks/Hazards/Discomforts

Stopping therapy

A potential negative effect of intermittent HAART is the development of virus resistance to the drugs employed. However, there is no indication from current therapy interruption that this is a significant risk in patients receiving PI-containing regimens (6,7 and M Dybul, personal communication). Furthermore, from a theoretical standpoint, since medications are stopped completely, and the drugs are, in general, rapidly cleared from the system (S. Piscitelli, personal communication), there is little reason to expect the development of resistance. This may be particularly true given the short off-drug periods since we have not observed a significant increase in HIV replication in reservoir sites, nor have we detected an increase in resistance to HAART in up to 1 year of short cycle intermittent therapy in the pilot study (M. Dybul, personal communication). Patients will be monitored closely for unexpected increases in viral load or decreases in CD4+ T-cell counts.

If patients have a significant rebound in plasma viremia, it is possible that they will experience a recurrence of the HIV prodromal syndrome including fever, fatigue, rash and aseptic meningitis. However, this generally occurs with very high levels of plasma viremia. Given the results of the pilot study, we do not anticipate this effect in many, if any, of the participants.

Study medications

All HAART medications can be associated with general constitutional symptoms of nausea, emesis, fatigue, fever, and arthralgia, as well as hypersensitivity reactions ranging from rash and pruritis to anaphylaxis and Stevens-Johnson syndrome. Side effects from HAART medications will be discussed as needed in keeping with standard clinical practice.

Study Procedures

Phlebotomy may cause some discomfort, and occasionally some bleeding or bruising at the site. On rare occasions, people faint while having blood drawn. Blood drawing will not exceed 450 milliliters (1 pint) over any 6 week period, however, the routine draws will constitute much smaller volumes.

Intervention or withdrawal criteria

1. Development of life-threatening infection or malignancy other than mucocutaneous Kaposi's sarcoma
2. Development of serious HIV-related illness
3. Patient's desire for early termination of participation, for any reason 4. Permanent discontinuation of all antiretroviral drugs
5. Patients on intermittent therapy who revert to continuous therapy
6. If it is felt by the principal investigator to be in the patient's best interest, even if the patient does not agree
7. Inability to tolerate procedures or study requirements
8. Becoming pregnant
9. Termination of study
10. As noted, failure of therapy will be determined by a viral load >1,000 copies/ml on 2 consecutive tests or > 10,000 copies/ml on a single, confirmed measurement) or a > 30% reduction from baseline (as determined by the mean of screening and enrollment values) or an absolute CD4+ T cell count < 100 cells/mm^3 in CD4+ T cell counts on 2 consecutive measurements. Failure will also be achieved if an individual develops an opportunistic infection (as determined by the 1993 US CDC criteria for AIDSdefining OIs). In the event of treatment failure, the HAART regimen will be changed in accordance with standards of care. For individuals in the intermittent group, treatment failure would not automatically require a switch in the patient's regimen. However, these decisions will be made by best clinical judgment with the best interest of the patient being the primary goal. If patients have virologic failure, genotypic and phenotypic resistance testing may be performed to assist in guiding therapy.

Data and safety monitoring

Although this is an open-label, randomized study, an independent Data Safety Monitoring Board (DSMB), appointed by the Office of the Clinical Director, NIAID, will oversee the study. The DBMS is appointed to assure the safety of subjects, to monitor the conduct of the protocol, and to monitor the validity and integrity of the data. The DSMB operates according to standard procedures approved by the Clinical Director.

The DSMB will include an expert in HIV, statistics, and a member from Uganda. The latter will be on an ad hoc basis. The DSMB will review safety data, including all SAEs and treatment failures on a bi-annual basis, at least yearly after 50% of patients have enrolled on each cohort. A mandatory conference call will occur if there is greater than a 30% incidence of failure in any arm after that arm has accrued more than 10 patients. There will be a universal stop rule for an intermittent arm if it has a failure rate more than 25% higher than the failure rate in the continuous arm.

Study Monitoring

Monitors under contract to the NIAID will visit participating clinical research sites to monitor all aspects of the study, with respect to current DHHS Code of Federal Regulations/ICH-GCP for compliance to the applicable guidelines. The objectives of a monitoring visit will be to verify the prompt reporting of all data points, including reporting of SAES; checking availability of signed

Informed Consent, and to compare individual subject records, CRFs and the source documents (supporting data, laboratory specimen records, and medical records to include physicians progress notes, nurses' notes, subjects'. hospital charts), to ensure protection of study subjects, compliance with the protocol, and accuracy and completeness of records. The monitors also will inspect clinical sites regulatory files to ensure that regulatory requirements (DHHS Code of Federal Regulations/ICH-GCP) are being followed. During the monitoring visits, the investigator (and/or designee) and other study personnel should be available to discuss the study.

The investigator will make study documents (e.g., consent forms, CRFs) and pertinent hospital or clinic records readily available for inspection by the local IRB, the site monitors, the NIAID for confirmation of the study data.

Data Review/Data Collection

Shared excel files will be maintained to include viral load, CD4+ T cell counts and abnormal safety labs. The excel files must be updated by JCRC weekly. There will be a conference call between the NIH PI and an investigator from JCRC at least twice per month to review patient status; in particular, all patients with a recent failure criteria must be discussed. There will be a study coordinator at JCRC whose sole responsibility will be to manage this study.

Case Report Forms (CRF) will be provided for each subject. Subjects will not be identified by name on the study CRFs. Subjects will be identified by the Patient Identification number and study identification number upon enrollment into the study.

All data on the CRFs must be legibly recorded in black ink. A correction should be made by striking through the incorrect entry with a single line and entering the correct information adjacent to it. The correction must be initiated and dated by the investigator or a designated, qualified individual. Any requested information that is not obtained as specified in the protocol should have an explanation noted on the CRF as to why the required information was not obtained.

Complete source documentation (test results, supporting data, laboratory specimen records, and medical records to include physicians progress notes, nurses' notes, subjects' hospital charts etc.) is required for every study subject for the entire duration of the study. Data reported on the CRFs must have a source document associated with it in order to
verify data being reported. If a source document is not available for a certain test it must be identified prior to starting this trial and documented on the CRF and in the study manual that a source is not available for tests procedures identified.

Adverse Event Reporting

An adverse event is any unfavorable or unintended change in body structure, body function or laboratory result associated temporally with the use of study treatment, whether or not considered related to the study treatment. Each adverse event will be graded according to the toxicity table. The US PI must be notified of all SAEs within 2 days. If an adverse event is not included in the toxicity table, the events will be graded according to the following:

Grade 1 (mild): transient or mild discomfort; no limitation in activity; no medical intervention/therapy required.

Grade 2 (moderate): mild to moderate limitation in activity; some assistance may be needed; no or minimal medical intervention required.

Grade 3 (severe): marked limitation in activity; some assistance usually required; medical intervention/therapy required.

Grade 4 (maximal/life threatening): extreme limitation in activity; significant assistance required.

Grade 5 (fatal): events which cause or contribute to the death of a subject.

When intensity changes occur more frequently than once a day, the maximum severity for the event should be listed. If the intensity category changes over a number of days, then these changes should be recorded separately, with inclusion of distinct onset and stop dates for each grade.

Serious Adverse Events

A serious adverse event includes any event, which is:
1.fatal or life-threatening;
2.leads to permanent disability;
3.requires or extends inpatient hospitalization; 4.a congenital anomaly;
5.an overdose of study drug with associated grade 3 or 4 event.

Life threatening refers to an adverse event, which at occurrence represented an immediate risk of death to the subject. An event which may have caused death had it occurred in a more severe form is not considered life threatening. Similarly, a hospital admission for an elective procedure is not considered a Serious Adverse Event.

The following explains (1) how information on adverse events that occur in patients participating on this protocol will be collected, monitored, and analyzed; and (2) what types of adverse events will be reported to the IRB in both the US and Uganda, and to the DSMB if appropriate.

(1) Information on adverse events is collected by nurse case managers, and other clinic staff and entered into a computerized database. The data are reviewed on an ongoing basis by the study coordinator and the principal investigator.

(2) The following types of serious adverse events will be reported to the IRB in writing within 7 days (for death or life threatening adverse events) and within 15 days for other events.

a. Death of a patient

b. Life-threatening events and or hospitalizations which are considered probably, possibly, or definitely related to the study drug or study procedures.

c. Events which cause permanent or significant disability/incapacity and are considered probably, possibly, or definitely related to the study drug or study procedures. d.Congenital

anomalies or birth defects.

e. All AIDS defining illnesses in patients will be reported to the IRB within 15 days of our having a working knowledge of the details.

f .All pregnancies occurring at any point within the trial will be reported to the IRB within 15 days of our having a working knowledge of the details. g.Any other event or condition regardless of grade which in the judgment of the investigator represents a reportable event in this category

The following types of adverse events will be reported to the IRB as part of the protocol continuing review.

a. All grade 3 and 4 toxicities regardless of relationship to study medication b.Admissions to hospitals resulting from causes unrelated to this study drug or study procedures Any other event or condition regardless of grade or relationship to study intervention which in the judgment of the investigator represents a reportable event in this category.

Duration of study

The duration of the study is 72 weeks. If a patient achieves a virologic failure endpoint, the patient will be followed until 30 days after they have achieved a plasma HIV RNA < 50 copies/ml or the CD4+ T cell count returns to baseline, depending on the failure criteria that were met. If a patient develops an 01, they will be followed for 30 days after resolution of that event. Patients who do not re-suppress to <50 copies/ml will be followed for 72 weeks or, 3 months after resuming continuous therapy, if continuous therapy is started within 2 months of the 72 week participation period.

Research Use of Stored Human Samples

Intended use of samples/specimens/data
Samples and data collected under this protocol may be used to study the effects of short cycle intermittent versus continuous HAART on viral load and other biochemical or histological indicators of HIV disease progression including genotypic and phenotypic antiretroviral resistance. Any other research or experimental treatments will be done under other protocols for which separate IRB review and approval will be obtained.

How samples/specimens/data will be stored
Samples will be secured in freezers accessible by authorized study personnel. Samples and data will stored by coded identification number assigned by the investigators or designee. Data will be kept in a password-protected data base management system. Only investigators or their designee will have access to the samples and data.

How samples specimens/data will be tracked
Samples will be tracked at the Joint Clinical Research center by use of a manual tracking sheet. Samples that are sent back to the NIH will be tracked using an electronic spread sheet with chain of custody signatures maintained at each transfer point and upon receipt at the final destination lab.

What will happen to samples/specimens/data at completion of the protocol
In the future, other investigators (both at NIH and outside) may wish to study these samples and

or/data. IN that case, IRB approval must be sought prior to any sharing of samples. Any clinical information shared about the sample with or without patient identifiers would similarly require IRB approval.

Circumstances that would prompt the PI to report to the IRB loss or destruction of samples/specimens/data
The NIH Intramural Protocol Violation definition related to loss of or destruction of samples (for example, due to freezer malfunction) will be followed in reporting to the IRB: The violation comprises the scientific integrity of the data collected for the study.

Any loss or unanticipated destruction of samples (for example, due to freezer malfunction) or data (for example, misplacing a printout of data with identifiers) will be reported to the IRB.

Benefits

Patients will be provided with viral load (USD 120) and lymphocyte subset counts (USD 30) at least every 6 weeks during study participation. The cost of these laboratory evaluations can exceed the cost of anti-retroviral medications and can be prohibitive for many patients (P. Mugyenyi, personal communication). For patients receiving intermittent therapy, there will be a 40-50% reduction in the cost of HAART drugs for the duration of their participation in the study. In addition, there may be an indirect benefit in providing important information for HIV-infected individuals, in particular, those living in the Southern Hemisphere.

Analysis of study

The sample size for this equivalency trial was determined using the method of Farrington and Manning*. For a one-sided equivalence test of proportions where the proportion of non-rebounding individuals in each group is 0.95, a sample size of 52 in each group has

81% power at an alpha level of 0.025, which will adjust to 0.05 for the comparisons of the control group with each of the 2 intermittent treated groups. The maximum allowable difference between these proportions that still results in equivalence is 0.15. Five additional patients have been added to each group to allow for an approximate 10% attrition of study individuals.

The test of equivalence of the proportions of non-rebounding individuals in each of the two intermittently treated groups with the proportion of those in the continuously treated group will be done by the Farrington and Manning test for equivalency.

The mean baseline levels, paired differences, and percent change in study measurements and regression slopes for the 2 intermittently treated groups will be compared with those of the continuously treated group by analysis of variance (ANOVA) with Dunnett's test where the ANOVA residuals are normally distributed. Otherwise, distributions with their median values for these measurements will be compared using the Kruskal-Wallis test with the Wilcoxon two-sample test; the adjustment of p values for multiple testing will be done using the Bonferroni method.

*Farrington, C.P. and Manning, G. Test statistics and sample size formulae for comparative

binomial trials with null hypothesis of non-zero risk difference or non-unity relative risk. Statistics in Medicine 1990, 9: 1447-1454.

Protection of human subjects

This protocol must receive the approval of the NIAID and JCRC Institutional Review Board prior to implementation. All participants must sign an informed consent form (see sample below). Patients will be fully counseled prior to entry into the study as to the potential **risks** of intermittent and continuous HAART. Because it is routine practice in the local community, patients who are unable to write their name to sign consents with a thumbprint. In this case, the counselor will certify that they have read the appropriate consent (Uganda or English) to the patient in addition to performing the regular counseling for informed consent. The confidentiality of all study participants will be protected in accordance with standard NIH procedures. Male and female patients will be enrolled without preference to gender.

Prevalence/Population Data

All HIV-1 infected women and men over the age of 18 who qualify based on the specific entry and exclusion criteria (see above) are welcome to participate in this study. It is hoped that the racial and ethnic character of the participants in this study will reflect, as closely as possible, the demographics of the HIV-1 epidemic in Uganda.

Inclusion of Children and Pregnant Women

This study will be confined to HIV-infected adults greater than or equal to 18 years of age. Although there are no data in Africa, there are clear differences in children in immune responses depending on the timing of infection, responses to ARV therapy in terms of kinetics and durability of response, perhaps as a result of complicating factors such as adherence. As a result, the effects of intermittent therapy in children are unknown. Studies are being planned by the ATN and PACTG in this country to help address specific issues in children regarding intermittent therapy. The management of HIV in pregnancy is also a specialized area and is beyond the scope of the present investigation.

References

1. CDC. *Morb Mortal Wkly Rep.* 1998;47:1-82.
2. Nataranjan V, et al. *Lancet.* 1999;353(9147):119-20.
3. Furtado MR, et al. *N Engl J Med.* 1999;340(21):1614-22.
4. Zhang L, et al. *N Engl J Med.* 1999;340(21):1605-13.
5. Davey R, et al. *Proc. Natl Acad Sci* 1999;96(26):15109-14.
6. Neumann A, et al. *AIDS.* 1999;13:677-683.
7. Garcia F, et al. *AIDS.* 1999;13:F79-F86.
8. Oritz GM, et al. *JClin Invest.* 1999;104:R13-R18.
9. Behrens G, et al. *AIDS.* 1999;13(10):F63-F70.
10. Miller KD, et al. *Lancet* 1998;351(9106):871-5.
11. Stephenson *J. JAMA.* 1998;279:641-2.
12. Ickovics J, et al. *J Clin Epidemiol.* 1997;50:385-91.
13. Stephenson *J. JAMA.* 1999;281:1069.
14. Deeks, S. G., et al. *AIDS* 1999;13: F35-43.
15. Lucas, G. M., et al. *Ann Intern Med* 1999; 131: 81-7.
16. Stephenson, J. *JAMA* 1999; 281,:1069.
17. Piot, P. et al. *Nature* 2001;410:968-73.
18. Dybul, M., et al. *Proc Natl Acad Sci, in press.*
